# CRISPR-Cas orthologues and variants: optimizing the repertoire, specificity and delivery of genome engineering tools

**DOI:** 10.1007/s00335-017-9697-4

**Published:** 2017-06-20

**Authors:** Alberto Cebrian-Serrano, Benjamin Davies

**Affiliations:** Wellcome Trust Centre for Human Genetics, University of Oxford, Oxford, OX3 7BN UK

## Abstract

Robust and cost-effective genome editing in a diverse array of cells and model organisms is now possible thanks to the discovery of the RNA-guided endonucleases of the CRISPR-Cas system. The commonly used Cas9 of *Streptococcus pyogenes* shows high levels of activity but, depending on the application, has been associated with some shortcomings. Firstly, the enzyme has been shown to cause mutagenesis at genomic sequences resembling the target sequence. Secondly, the stringent requirement for a specific motif adjacent to the selected target site can limit the target range of this enzyme. Lastly, the physical size of Cas9 challenges the efficient delivery of genomic engineering tools based on this enzyme as viral particles for potential therapeutic applications. Related and parallel strategies have been employed to address these issues. Taking advantage of the wealth of structural information that is becoming available for CRISPR-Cas effector proteins, Cas9 has been redesigned by mutagenizing key residues contributing to activity and target recognition. The protein has also been shortened and redesigned into component subunits in an attempt to facilitate its efficient delivery. Furthermore, the CRISPR-Cas toolbox has been expanded by exploring the properties of Cas9 orthologues and other related effector proteins from diverse bacterial species, some of which exhibit different target site specificities and reduced molecular size. It is hoped that the improvements in accuracy, target range and efficiency of delivery will facilitate the therapeutic application of these site-specific nucleases.

## Introduction

The discovery and application of Clustered Regularly Interspaced Short Palindromic Repeats (CRISPR) and CRISPR-associated (Cas) systems for genetic modification have revolutionized biomedical research in just a few years. The CRISPR-Cas9 system has proven itself to be a robust genome editing tool in mammalian cells and animal models, and has rapidly shown its great potential in diverse fields such as functional genomics, genome-wide screening studies, therapeutic gene therapy and agricultural applications. The technology based on CRISPR-Cas9 has surpassed other nucleases that preceded it, such as the zinc finger nucleases (ZFNs) and transcription-activator-like effector nucleases (TALENs) in terms of simplicity, efficiency and amenability to multiplexing, becoming the most broadly implemented approach for genome engineering today.

CRISPR-Cas systems are natural RNA-guided adaptive immune systems of bacteria and archaea that provide sequence-specific resistance against viruses or other invading genetic material. This immune-like response has been divided into two classes on the basis of the architecture of the effector module responsible for target recognition and the cleavage of the invading nucleic acid (Makarova et al. 2015). Class 1 comprises multi-subunit Cas protein effectors and Class 2 consists of a single large effector protein. Both Class 1 and 2 use CRISPR RNAs (crRNAs) to guide a Cas nuclease component to its target site where it cleaves the invading nucleic acids. Due to their simplicity, Class 2 CRISPR-Cas systems are the most studied and widely applied for genome editing.

## CRISPR-Cas9 system

Cas9, the nuclease, is active when it forms a complex with two naturally occurring RNA species, the tracrRNA and the crRNA (Jinek et al. 2012). The first 20 nucleotides of the crRNA sequence define the specificity of the nuclease, which occurs by complementary base pairing with the target sequence within genomic DNA. Once activated, the nuclease generates a double-strand break (DSB) at the target site. Cas9 uses two distinct active sites, RuvC and HNH, generating site-specific nicks on opposite DNA strands (Gasiunas et al. 2012; Jinek et al. 2012). By simply specifying the targeting sequence of the crRNA, one can direct the CRISPR-Cas9 system to the appropriate genomic target site. An additional requirement for Cas9-mediated genome cleavage is the presence of a short and conserved protospacer adjacent motif (PAM) flanking the genomic target site. Functionality in mammalian cells was rapidly demonstrated and the native bacterial system was further simplified into a two-component system, with the crRNA and tracrRNA fused together to form a single-guide RNA (sgRNA) (Cho et al. 2013; Cong et al. 2013; Jinek et al. 2013; Mali et al. 2013a).

CRISPR-Cas9 systems promote genome editing by inducing a DSB at a target genomic loci, which is quickly acted upon by the cell’s DNA repair machinery. The generated ends of DNA can be religated by non-homologous end joining (NHEJ), a process known to be quite precise (Bétermier et al. 2014) but which can also introduce indel mutations at the DSB site (Lieber 2010), especially when the nucleases are active in the cell for a prolonged period. Alternatively, regions of homology flanking the DSB can lead to a process known as microhomology mediated end joining (MMEJ), also introducing indel mutations at the target site (Sinha et al. 2017). These indel mutations provide a means of disrupting protein coding or other functional DNA sequences. An additional pathway, homology directed repairs (HDR), can be adopted by the cell to repair the lesion accurately if homologous DNA sequences are present (Greene 2016). The HDR repair pathway enables intentional replacement of endogenous genomic sequence with information on a homologous donor molecule, presented to the cell. These templates can be delivered as single stranded oligodeoxynucleotide (ssODN) harbouring desired nucleotide changes (Cong et al. 2013) or as conventional double-stranded DNA targeting constructs (Chu et al. 2016), in both cases with regions of homologous sequence flanking the sequence change.

Among the different bacteria harbouring CRISPR-Cas systems, the human pathogen *Streptococcus pyogenes* was one of the first bacteria in which CRISPR type II loci were characterized (Deltcheva et al. 2011). Furthermore, it was SpCas9 that was first used to cleave and edit a specific region of the genome of a human cell (Cho et al. 2013; Cong et al. 2013; Jinek et al. 2013; Mali et al. 2013a). In the following months, many groups applied SpCas9-mediated genome editing successfully across a broad range of animal models, including yeast, nematode, fruit fly, zebrafish, mouse, pig, rabbit and monkey (reviewed by Sander and Joung 2014).

## SpCas9 modification: SpCas9 nickase

**Fig. 1:**
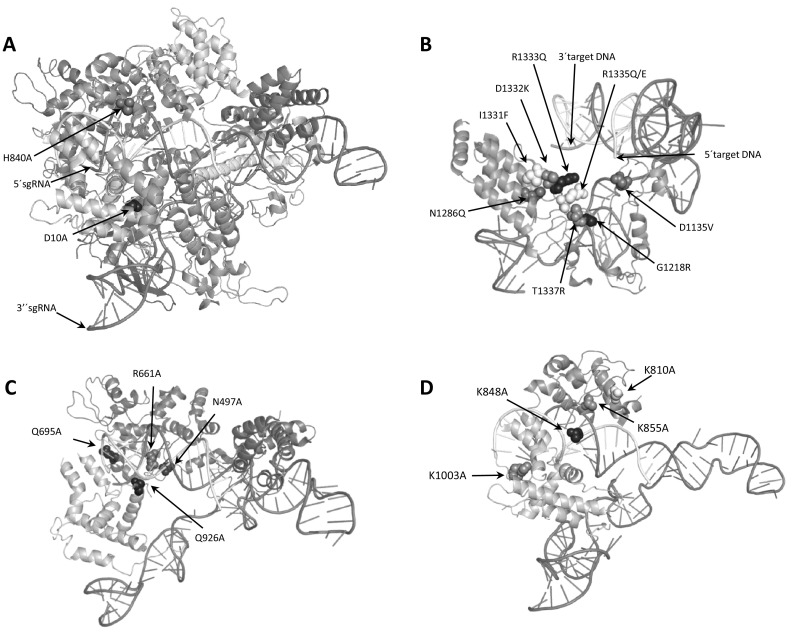
Position of mutagenized residues in SpCas9. **a** Crystal structure of SpCas9 in complex with sgRNA and target DNA (PDB ID 4OO8). The position of the catalytic residues responsible for the HNH (Asp-10) and the RuvC (His-840) nuclease activity, which are mutated in the D10A and H840A nickases, are shown in *black* and *grey* respectively. **b** Detail of the PAM interaction domain in complex with guide RNA and target DNA showing the position of the key residues mutated in variant Cas9 with altered PAM specificities and how they are mutated. **c** and **d** Detail of the target interaction domain in complex with guide RNA and target DNA showing the position of the key residues mutated in SpCas9-HF1 (**c**) and eSpCas9 (**d**) and how they are mutated. Residue Arg-1060, mutated in eSpCas9 (1.0) and (1.1), is not annotated in the crystal structure. The 20 bp target DNA is shown in *white* and the sgRNA is shown in *red*. (Color figure online)

Despite the simplicity and high levels of mutagenesis reported with SpCas9, concerns about specificity were quickly raised; it was found that the crRNA could tolerate certain mismatches to the DNA target and could cleave and thus mutagenize the genome at these mismatched sequences—so-called off-targets (Cradick et al. 2013; Fu et al. 2013). Although the real importance of off-target effects is still under study (Iyer et al. 2015), these specificity limitations would clearly complicate potential therapeutic applications and produce a source of variability in biological studies. With the objective of reducing potential off-target effects without decreasing efficiencies, Cas9 variants were developed which had each of the two nuclease domains silenced by a point mutation of a key catalytic residue (D10A for HNH and H840A for RuvC) (Fig. 1a; Table 1) (Cong et al. 2013; Mali et al. 2013b; Ran et al. 2013). These so-called nickases (nSpCas9) retained the specificity of action but only cleaved a single strand of the DNA. This approach benefits from the fact that individual nicks in the genome are repaired with high fidelity (Dianov and Hübscher 2013), while nicking of both DNA strands by a pair of opposite orientated Cas9 nickases in close proximity would lead to a DSB that would then be processed by the NHEJ or HDR activities of the cell. Although this approach clearly reduces the number of possible target sites available within the genome, the method has been used successfully to facilitate efficient genome engineering with minimal off-target effects in mammalian cells and mouse zygotes (Cho et al. 2014; Cong et al. 2013; Ran et al. 2013; Shen et al. 2014).

**Table 1:** Characteristics of *Streptococcus pyogenes* Cas9 variants used for genome editing

CRISPR-Cas9 variants	Size (aa)	PAM sequence	Target length	PBD ID	References
*Streptococcus pyogenes * Cas9	1368–1424	NGG	20 nt	4OO8 (Nishimasu et al. 2014), 4UN3 (Anders et al. 2014), 4CMP (Jinek et al. 2014), 4ZT0 (Jiang et al. 2015) and 5F9R (Jiang et al. 2016)	Cong et al. (2013); Jinek et al. (2013; Mali et al. (2013a)
*Streptococcus pyogenes * Cas9 nickase	1368–1424	NGG	20 nt	4ZT9 (Jiang et al. 2015)	Cong et al. (2013); Mali et al. (2013b); Ran et al. (2013); Cho et al. (2014); Shen et al. (2014), Eggenschwiler et al. (2016); Osborn et al. (2015); Wu et al. (2016); Sakuma et al. (2016); Lee and Lloyd (2014); Mianné et al. (2016); Bialk et al. (2015); Ghezraoui et al. (2014); Miyaoka et al. (2016); Richardson et al. (2016)
Dimeric dCas9-FokI	1817	NGG	20 nt		Guilinger et al. (2014); Tsai et al. (2014); Aouida et al. (2015); Hara et al. (2015); Pan et al. (2016); Terao et al. (2016)
*Streptococcus pyogenes * Cas9 Variant VQR	1372	NGAG	20 nt	5FW1 (Anders et al. 2016), 5B2R (Hirano et al. 2016b)	Kleinstiver et al. (2015b)
*Streptococcus pyogenes * Cas9 Variant EQR	1372	NGAG	20 nt	5FW2 (Anders et al. 2016), 5B2S (Hirano et al. 2016b)	Kleinstiver et al. (2015b)
*Streptococcus pyogenes * Cas9 Variant VRER	1372	NGCG	20 nt	5FW3 (Anders et al. 2016), 5B2T (Hirano et al. 2016b)	Kleinstiver et al. (2016)
*Streptococcus pyogenes * Cas9 Variant D1135E	1372	NAG and NGA	20 nt		Kleinstiver et al. (2016)
*Streptococcus pyogenes * Cas9 Variant QQR1	1372	NAAG	20 nt		Anders et al. (2016)
High-fidelity *Streptococcus pyogenes * Cas9 SpCas9-HF1	1368	NGG	20 nt		Kleinstiver et al. (2016)
“Enhanced specificity” *Streptococcus pyogenes * Cas9: SpCas9 (K855A), eSpCas9 (1.0), and eSpCas9 (1.1)	1424	NGG	20 nt		Slaymaker et al. (2016)

Using paired nSpCas9 to induce large deletions, up to 1 kb deletions have been reported in human cells without observing unwanted translocations (Cho et al. 2014). More recently, efficient bi-allelic targeting of a silent locus and a single nucleotide substitution has been described using nSpCas9 in patient derived induced pluripotent stem (iPS) cells (Eggenschwiler et al. 2016) and fibroblasts (Osborn et al. 2015). Also in human iPS cells, Wu et al. reported a GFP insertion by nSpCas9 (Wu et al. 2016). Addressing an episomal target, nSpCas9 was used successfully to target multiple Hepatitis B virus closed circular DNA in HepG2 cells without apparent off-target mutations (Sakuma et al. 2016). In two studies performed in vivo in mouse zygotes, knock-in manipulations, generating a floxed allele (Lee and Lloyd 2014) and a single nucleotide substitution (Mianné et al. 2016), were successfully performed by the injection of nSpCas9 as mRNA with paired sgRNA guides and ssODN donor templates.

Side-by-side analysis of mutation efficiencies using either wild-type SpCas9 or a paired nickase have revealed conflicting results. An initial study reported higher activity for the wild-type enzyme than the nickase on a mutant eGFP gene (Bialk et al. 2015), although whether this result would hold up across many different loci in unclear. In a further comparison, oncogenic chromosome translocations were induced by either wild-type SpCas9 enzymes or paired nSpCas9 combinations with lower translocation frequency reported for paired nSpCas9 (Ghezraoui et al. 2014). In the results, the deletions observed at the translocation junctions generated with nSpCas9 were significantly longer than those generated from wild-type Cas9, presumably due to processing of large overhangs generated by the nickase enzyme. With the aim to characterize HDR/NHEJ ratios, a comparison between single and dual nSpCas9 systems found that single, dual and tandem (Cas9-D10A plus Cas9-H840A) nSpCas9 can induce more HDR than NHEJ in HEK293 cells (Miyaoka et al. 2016). In agreement, nCas9 was found to promote HDR and minimize NHEJ in a traffic light reporter assessment of DNA repair fates in this same cell type (Osborn et al. 2015). Potentially, this might also be due to the staggered overhangs which inevitably occur following the action of paired nickases which are considered to be more recombinogenic than the blunt ends generated by the wild-type Cas9. Future work must examine whether these observations hold true across multiple genomic loci and cell types and the analysis of knock-in efficiencies might cast light on how the overhangs can be designed to enhance HDR repair.

Recently, concerns about the use of paired Cas9 nickase for genome editing has been raised (Richardson et al. 2016). Using single nickase ribonucleoprotein electroporation, single nicks induced by either Cas9-D10A or Cas9-H840A were able to stimulate HDR (~10%) in cells when provided with the ssDNA, and also silenced a BFP reporter gene, probably by inducing error-prone NHEJ. Similarly, single nicks have been shown to promote efficient HDR via an alternative HDR pathway (Davis and Maizels 2014), with little accompanying mutagenic end joining observed, which is of course ideal for genome engineering applications. It is as yet unclear how universal these phenomena are, and it is becoming clear that both the cell type and the delivery route of the nuclease or nickase may play an important role in determining repair outcome (Kim et al. 2014). Certainly, the suggestion that single nicks can be recombinogenic undermines the argument that these lesions within the genome are accurately repaired, thus questioning the validity of the paired nickase approach for reducing off-target mutagenesis.

## FokI-fused catalytically inactive Cas9

One of the main differences between ZFNs and TALENs endonucleases and CRISPR-Cas based system concerns specificity and this is clearly a result of its mechanism of action. As previously discussed, Cas9 is a monomeric nuclease which acts when coupled to its guiding sgRNA to produce a DSB at its target site. In contrast, the nuclease domain of ZFNs and TALENs requires dimerization for activity and subsequently these dimeric nucleases cleave DNA only when two simultaneous, adjacent monomer-binding events take place. The likelihood of dimeric off-target binding events is considerably smaller than the likelihood of a monomeric off-target binding event and thus specificity is markedly improved. With these principles in mind, SpCas9 has been adapted to a dimeric nuclease with a similar mode of binding, potentially matching the specificities of ZFNs and TALENs, although similar to the double nickase approach, the number of potential genomic target sites for these Fok1-dimeric nucleases is reduced. SpCas9 was rendered catalytically inert by mutation of both nuclease domains, creating what is known as a “dead-Cas9” (dCas9) which was then fused to the Fok1 nuclease domain (fCas9) (Aouida et al. 2015; Guilinger et al. 2014; Tsai et al. 2014). Functionality of this enzyme in achieving mutagenesis of target sites in human cells was demonstrated and these reports also confirmed the specificity of the approach—cleavage activity of fCas9 depends on the simultaneous binding of two guide sgRNAs to their genomic DNA targets with a defined spacing and orientation (15–25 base pairs apart). The fCas9 approach was used to correct the phenylalanine hydroxylase gene in COS-7 cells (Pan et al. 2016). fCas9 has also been shown to be suitable for the generation of knockout mice by microinjection of zygotes with fCas9 mRNA and two sgRNAs spaced 14–19 bases apart (Hara et al. 2015). This study reported similar genome editing efficiencies than those observed using wild-type SpCas9 and nSpCas9. To further simplify the production of mutant mice, genetically modified mice were obtained using chemically synthesized crRNA and tracrRNA plus nSpCas9 or fCas9 by microinjection into fertilized eggs (Terao et al. 2016). A side-by-side comparison using fCas9, nSpCas9 and wild-type SpCas9 to target six human genes showed average indel frequencies of 14.9, 20.6 and 28.2%, respectively (Guilinger et al. 2014), suggesting a drop in efficiency with these Cas9 variants.

## Engineered Cas9 variants with novel PAM specificities

One of the key factors that determine CRISPR-Cas specificity is the nature of the PAM sequence. In the case of SpCas9, a DSB is produced when a NGG sequence (canonical PAM) lies immediately 3′ of the target DNA sequence. However, it has been reported that alternative PAM sequences (3′ NAG and NGA) can, to an extent, be recognized by the SpCas9–sgRNA complex (Kleinstiver et al. 2015b; Zhang et al. 2014), potentially increasing the likelihood of off-target mutagenesis. The requirement for a 3′ NGG PAM is, however, considered quite stringent and, depending on the primary sequence of DNA, this might limit the sequences that can be addressed, particularly in A/T-rich regions of the genome. One potential solution to address targeting range limitations would be to engineer Cas9 variants with novel PAM specificities. Kleinstiver et al. explored the possibility of rendering SpCas9 sensitive to alternative PAM sequences by introducing mutations into the PAM-interacting domains of wild-type SpCas9 (Kleinstiver et al. 2015b). SpCas9 variants were successfully engineered which recognize NGCG (variant VRER, D1135V/G1218R/R1335E/T1337R), NGAG (variant VQR, D1135V/R1335Q/T1337R) or NGAG (variant EQR, D1135E/R1335Q/T1337R) (Fig. 1b; Table 1), previously inaccessible sites, and these enzymes showed similar (or better) genome-wide specificities compared to wild-type SpCas9 in human cells and zebrafish embryos. In addition, a D1135E mutation in SpCas9 was found to improve the PAM recognition and specificity of SpCas9, decreasing off-target mutagenesis with non-canonical NAG and NGA PAMs and/or mismatched target sites. A NAAG specific SpCas9 variant (variant QQR1, G1218R/N1286Q/I1331F/D1332K/R1333Q/R1335Q/T1337R) (Fig. 1b; Table 1) was engineered and tested in vitro, revealing a slower cleavage activity than that of wild-type SpCas9 (Anders et al. 2016).

## DNA-binding domain fusions

Cas9 has also been fused to other programmable DNA-binding domains, such as Zinc Finger binding domains and TALE domains, designed against sequences lying downstream of the Cas9 target site. By tethering the Cas9 in this sequence-specific manner, the requirement for the 3′ NGG PAM was partially overcome and cleavage of alternative PAM sequences was achieved (Bolukbasi et al. 2015). The same study combined this tethering idea with Cas9 mutations that compromised the key PAM recognition residues (Arg1333 and Arg1335). These attenuated Cas9-programmable DNA-binding domain (Cas9-pDBD) nucleases showed improved precision as judged by deep sequencing of previously characterized off-target sites for 3 genomic target sites. An unbiased assessment of off-target mutagenesis also revealed no new sites are generated by the Cas9-pDBD fusions (Bolukbasi et al. 2015).

## Engineering enzymes with increased fidelity—SpCas9-HF1/eSpCas9

Application of genome engineering in biomedicine is a major challenge and for successful clinical use, off-target mutagenesis needs to be eliminated on a genome-wide scale. One recently explored approach raised the idea of redesigning the DNA-binding domain of SpCas9 to reduce non-specific DNA contacts, thus increasing specificity. Studies of the crystal structure of SpCas9–sgRNA–target DNA complex have concluded that the SpCas9–sgRNA complex might possess more energy than is needed for optimal recognition of its intended target DNA site, which might then be responsible for off-target binding activity (Kleinstiver et al. 2016; Slaymaker et al. 2016). With this hypothesis in mind, 15 SpCas9 variants with residue substitutions in positions that form hydrogen bonds with the DNA backbone were constructed and the variant enzymes characterized for on and off-target activity (Kleinstiver et al. 2016). One of the SpCas9 variants with four residues substitution showed similar on-target efficiency, with at least 70% of the activity observed with native SpCas9, but exhibited considerably higher genome-wide specificity (SpCas9-HF1, N497A/R661A/Q695A/Q926A) (Fig. 1c; Table 1), reducing nearly all genome-wide off-target effects to near undetectable levels. In addition, this study demonstrated that the introduction of substitutions at other non-specific DNA contacting residues into the SpCas9-HF1 protein (similar to the above mentioned D1135E mutation) could further reduce some of the residual off-target activity that prevail for certain sgRNAs. In a complementary approach, Slaymaker et al. suggested that positively charged residues positioned between the HNH, RuvC, and PAM-interacting domains in SpCas9, in the so-called non-target strand groove, are involved in stabilizing the non-target strand of the target DNA (Slaymaker et al. 2016). Thus, a way to reduce the energy of the non-target strand binding would be to neutralize the positive charges positioned in this groove. After the characterization of 31 SpCas9 mutants, three were identified with similar efficiency than wild-type SpCas9 (SpCas9-K855A, eSpCas9 [1.1] [K848A/K1003A/R1060A] and eSpCas9 [1.0] [K810A/K1003A/R1060A]) (Fig. 1d; Table 1). The off-target assessment showed that two of the variants, SpCas9-K855A and eSpCas9 (1.1), exhibited a genome-wide reduction in off-target cleavage without generating any new off-target sites. Both studies (Kleinstiver et al. 2016; Slaymaker et al. 2016) reinforce the idea that mutations designed to weaken interactions in the SpCas9–sgRNA–target DNA complex can lead to a considerable improvement in specificity. It will be interesting to see whether, when applied in a variety of different experimental settings, the activity of these engineered enzymes at their designated targets matches that of the unmodified enzyme at all genomic target sites.

Modifications in the sgRNA may also increase the target specificity of the CRISPR-Cas9 system. A simple strategy of truncating the sgRNA sequence at the 5′ end with shorter regions of target complementarity has been reported with a reduction of off-target mutagenesis at some loci (Fu et al. 2014). As an alternative approach, the addition of two extra guanine nucleotides at the 5′ end of sgRNA has been shown as a factor that can affect mutation frequencies both on- and off-target (Cho et al. 2014). This manipulation of the sgRNA, advantageous for their efficient transcription by the T7 polymerase, was shown to decrease off-target site cleavage substantially (Kim et al. 2014; Kim et al. 2015). However, lower on-target mutagenesis efficiency was reported for certain target sites (Cho et al. 2014).

## Therapeutic delivery: size constraints

In addition to accuracy, for a realistic chance of applying genome editing tools in a therapeutic context, an efficient delivery mechanism must be assured. Adeno-associated virus (AAV) vectors are one of the most widely used delivery mechanisms for gene therapy (Naldini 2015). However, the cargo size of AAV (around 4.7 kb) is a limitation for packaging the SpCas9 (4.2 kb) and its promoter driven sgRNA in an “all in one” vector.

Structural studies of SpCas9 revealed a potential redundancy with the recognition lobe of the protein and thus a candidate region for shortening the protein, facilitating its therapeutic delivery. A deletion mutant (Δ175–307) was generated which did indeed retain nuclease activity, however overall activity was reduced by half which was a considerable price to pay for a saving of only 133 amino acids (Nishimasu et al. 2014). An alternative strategy for reducing the size of SpCas9 involved the separation of the recognition lobe from the nuclease lobe into two separate vectors. When used in combination with the sgRNA, a functional CRISPR-Cas9 nuclease was reconstituted, although activity as measured by indel mutagenesis in mammalian cells was reduced compared to the native enzyme (Wright et al. 2015). A similar split Cas9 approach, which used a dimerization domain to facilitate enzyme reconstitution, demonstrated similar functionality but also reported lower rates of mutagenesis (Zetsche et al. 2015b). Trans protein splicing using the intein–extein system provided a more robust solution of delivering, effectively, the native enzyme, with the two lobes of SpCas9 encoded on separate vectors (Truong et al. 2015). Indeed, successful packaging into recombinant AAV and delivery into cells in vitro was shown to achieve mutagenesis. Interestingly, there was sufficient capacity within the two recombinant viruses to include a homology template. Thus, delivery of site-specific nucleases together with a therapeutic template could be achieved in two modestly sized viral vectors.

## Cas9 orthologues

**Fig. 2:**
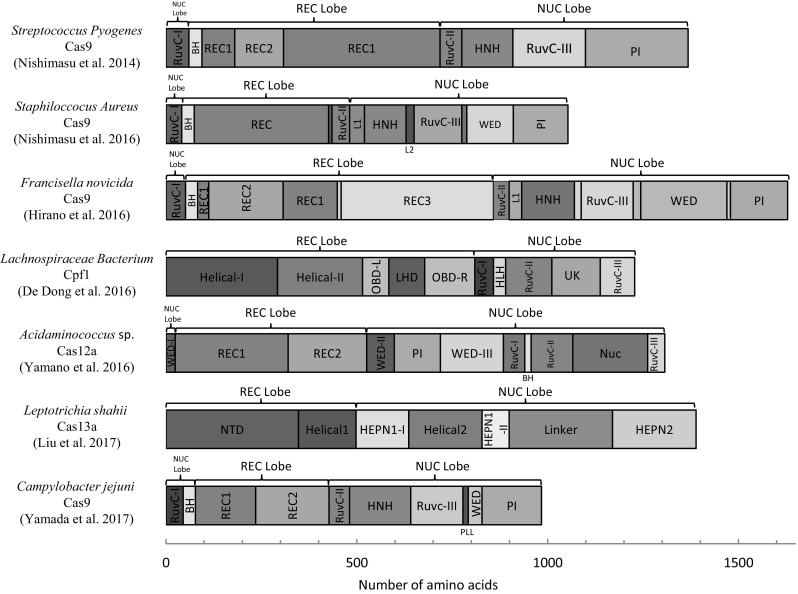
Domain structure of CRISPR-Cas effector orthologues. *REC* recognition domain, *NUC* Nuclease domain, *PI* PAM interaction domain, *BH* bridge helix domain, *L1 and L2* linker 1 and 2, *WED* wedge domain, *OBD* oligonucleotide-binding domain, *LHD* looped-out helical domain, *UK* Unknown function domain, *NTD* N-terminal domain, *PLL* phosphate lock loop

Another route to solving the delivery problem of the CRISPR-Cas9 components is to consider alternative CRISPR effectors from other bacterial and archeal species which are smaller in size (Fig. 2). Indeed, orthologous enzymes from other bacteria have been investigated and applied in mammalian systems, including the mouse zygote (Table 2). Interesting, as explored below, these orthologous enzymes show different substrate specificities, frequently recognizing a different length of target sequence and a different PAM. Thus, the use of Cas9 orthologues or other RNA-guided nucleases may not just provide a solution to the size limitation of using SpCas9 but might also widen the range of potential sequences that can be targeted.

**Table 2:** Characteristics of CRISPR-Cas effector orthologues used for genome editing

Species and effectors	Class and type	Size (aa)	PAM sequence	Target length	PBD ID	Cell line/organism targeted	References
*Staphylococcus aureus* Cas9	Class II type II	1,053	NNGRRT	20 to 24 nt	5AXW and 5CZZ (Nishimasu et al. 2015)	Human cells and mice (in vivo delivered by AAV delivery)	Ran et al. (2015)
						Mouse zygotes	Zhang et al. (2016)
						Mouse (in vivo delivered by AAV)	Kaminski et al. (2016)
						Human hematopoietic stem and progenitor cells	Ye et al. (2016)
						HEK293FT cells	Nishimasu et al. (2015)
*Staphylococcus aureus* Cas9 Nickase		1,053	NNGRRT	24 nt		HEK293T cells	Friedland et al. (2015)
*Staphylococcus aureus* KKH Cas9 variant		1,053	NNNRRT	21 nt		U2OS cells	Kleinstiver et al. (2015a)
*Streptococcus thermophilus1* Cas9	Class II type II	1,122	NNAGAAW	19 to 20 nt		HEK293FT cells	Cong et al. (2013)
						HEK293T cells	Esvelt et al. (2013)
						Mouse zygotes	Fujii et al. (2016)
						HEK293T cells	Muller et al. (2016)
*Streptococcus thermophilus*3 Cas9	Class II type II	1,393	NGGNG	19 nt		HEK293T cells	Xu et al. (2015)
						HEK293T cells	Muller et al. (2016)
						HEK293T cells	Glemzaite et al. (2015)
*Neisseria meningitidis* Cas9	Class II type II	1,109	NNNNGATT	23, 24 nt		Human induced pluripotent stem and HEK293FT cells	Hou et al. (2013)
						HEK293T cells	Esvelt et al. (2013)
						HEK293T cells	Lee et al. (2016)
*Francisella novicida* Cas9	Class II type II	1,629	NGG	22 nt	5B2O and 5B2P (Hirano et al. 2016a)	Mice zygotes	Hirano et al. (2016a)
*RHA Francisella novicida* Cas9 variant	Class II type II	1632	YG	22 nt	5B2Q (Hirano et al. 2016a)	Mice zygotes	Hirano et al. (2016a)
*Treponema denticola* Cas9	Class II type II	1,423	NAAAAN	20 nt		HEK293T cells	Esvelt et al. (2013)
*Acidaminococcus* Cas12a (Cpf1)	Class II type V	1,308-1,310	TTTV	23, 24 nt	5KK5 (Gao et al. 2016) 5B43 (Yamano et al. 2016)	Human HEK293FT cells	Zetsche et al. (2015a)
						N2a mouse neuroblastoma cells	Toth et al. (2016)
						Mice zygotes	Watkins-Chow et al. (2017)
						HEK293T cells	Kim et al. (2016a)
						HEK293T cells	Kim et al. (2017b)
						Mice zygotes	Kim et al. (2016b)
						Mice zygotes	Hur et al. (2016)
*Lachnospiraceae* Cas12a (Cpf1)	Class II type V	1,228	TTTV	23, 24 nt	5ID6 (Dong et al. 2016)	Human HEK293FT cells	Zetsche et al. (2015a)
						N2a mouse neuroblastoma cells	Toth et al. (2016)
						HEK293T cells	Kim et al. (2016a)
						HEK293T cells	Kim et al. (2017b)
						Mice zygotes	Kim et al. (2016b)
*Leptotrichia buccalis* Cas13a (C2c2)	class II type VI	1,159	N/A	N/A		Hela cell (extracts)	East-Seletsky et al. (2016)
*Leptotrichia shahii* Cas13a (C2c2)	Class II type VI	1,389	N/A	N/A	5WTJ and 5WTK (Liu et al. 2017)	*E. Coli*	Abudayyeh et al. (2016)
*Campylobacter jejuni* Cas9	Class II type II	984	NNNNACAC or NNNNRYAC	22 nt		HEK293T cells Mouse muscle and retinal pigment epithelium cells	Kim et al. (2017a)

N/A—Cas13a enzymes are yet to be applied in mammalian cells and as such, the functional target length and characteristics remains unclear

To explore Cas9 orthologues, the sequence databases of bacterial genome sequences can be mined for Cas9-like enzymes. Ran et al., used this approach and screened over 600 Cas9 sequences and discovered that they cluster into groups of either approximately 1350 amino acids (of which SpCas9 is a member) or a significant shorter 1000 amino acid group (Ran et al. 2015). The following section reviews orthologues with which appreciable activity has been demonstrated in mammalian systems.

### Staphylococcus aureus Cas9

*Staphylococcus aureus* (SaCas9) was found to have similar levels of gene targeting compared to SpCas9 (Ran et al. 2015). This enzyme targets sequences between 21 and 24 bp in length and requires a 3′ NNGRRT PAMs sequence. The shorter size of SaCas9 was compatible with packaging into an all-in-one AAV vector, which was shown to mediate efficient editing in vivo when delivered to mice (Kaminski et al. 2016; Ran et al. 2015). Further highlighting the therapeutic potential of SaCas9, efficient ex vivo genome editing of hematopoietic stem and progenitor cells was demonstrated by AAV delivery (Ye et al. 2016). SaCas9 has also revealed good levels of specificity with some improvement on SpCas9 in a side-by-side comparison (Friedland et al. 2015; Ran et al. 2015). In attempts to further restrict off-target mutagenesis, the nickase approach has also been applied to SaCas9, and interestingly the SaCas9 nickase, along with two sgRNA expression cassettes could be packaged into a single AAV vector (Friedland et al. 2015).

Whether the apparent increase in specificity reported for SaCas9 relates to the requirement for a longer PAM sequence remains to be explored. Clearly, the longer PAM sequence presents some disadvantages as the range of putative target sites would be reduced in comparison with the SpCas9 which recognizes a more simple and thus commonly occurring sequence. Perhaps to address this limitation, the PAM specificities of SaCas9 have also been manipulated by mutagenesis of the SaCas9 and a variant recognizing a NNNRRT PAM motif (variant E782K/N968K/R1015H, KKH SaCas9) has been reported which shows similar efficiency and specificity as the wild-type SaCas9 (Kleinstiver et al. 2015a). Although SaCas9 is a smaller enzyme than SpCas9 and thus may be more amenable for therapeutic delivery as a single virus, structural analysis of the SaCas9 has also allowed the design of a split-enzyme, which could potentially allow further efficiency improvements for the delivery of this nuclease (Nishimasu et al. 2015).

SaCas9 has also been applied to the fertilized zygote for generating genetically modified mice. Both single and multiplex gene disruptions were demonstrated and the generation of a knock-in modification (Flag tag insertion) was efficiently achieved (Zhang et al. 2016). Similar efficiencies between SaCas9 and SpCas9 were seen in a side-by-side comparison at two genomic targets with no difference in mosaicism observed, suggesting similar kinetics and activity for both SaCas9 and SpCas9. Mosaicism is a phenomenon described already with SpCas9 (Yen et al. 2014), whereby nuclease activity persists following the first round of cell division leading to allele complexity.

### Streptococcus thermophilus Cas9

*Streptococcus thermophilus* played a central role in the early history of CRISPR-Cas. It was in this bacterium that CRISPR-Cas9 was first recognized as a prokaryote immune system (Barrangou et al. 2007). Modified for mammalian expression, *Streptococcus thermophilus* LMD-9 Cas9 encoded by the CRISPR1 locus (St1Cas9) was found to show cleavage activity in mammalian cells (Cong et al. 2013) and was also selected on the basis of showing robust rates of homologous recombination, when compared against two other orthologues from *Neisseria meningitides, Treponema denticola* and SpCas9 (Esvelt et al. 2013). A direct comparison of St1Cas9 against SpCas9 and SaCas9 in two human loci, however, concluded a lower level of activity (Ran et al. 2015). Interestingly, St1Cas9 was found to require a more complex PAM sequences for activity (Table 2) and the position of cleavage with respect to the PAM was found to be far more heterogeneous than for other orthologues (Ran et al. 2015). St1Cas9 has also been successfully applied in the mouse zygote by microinjection for the generation of genetically modified mice harbouring both indel mutations as well as knock-in alleles (Fujii et al. 2016).

The related Cas9 from the CRISPR3 locus (St3Cas9) was also found to be active in human cells (Xu et al. 2015), and a side-by-side comparison with St1Cas9, St3Cas9 and SpCas9 revealed robust cleavage efficiencies across two genomic loci (Muller et al. 2016). This study also examined off-target mutagenesis and found the two StCas9s to be more accurate than SpCas9. The necessity for more complex PAM sites (NNAGAAW and NGGNG for St1Cas9 and St3Cas9, respectively) potentially contributes to this observation, similar to the observations with SaCas9. Direct delivery of St3Cas9 protein plus crRNA-tracrRNA complex into HEK293T cells was shown to be a reliable strategy for targeting human cells in vitro without the necessity for cloning procedures (Glemzaite et al. 2015).

### Neisseria meningitidis Cas9 (NmCas9)

The Cas9 of *Neisseria meningitidis* (NmCas9) has been explored as a potential alternative to StCas9, being smaller in length than SpCas9 and with a stringent PAM sequence recognition (5′-NNNNGATT-3′) (Table 2) (Zhang et al. 2013; Fonfara et al. 2014). NmCas9 became part of the Cas9 toolbox after studies demonstrated the ability of NmCas9 to induce homologous recombination in human cells (Esvelt et al. 2013; Hou et al. 2013). NmCas9 was found to recognize a 24 nt protospacer, which, together with its more complex PAM, might imply a high level of specificity. Indeed side-by-side comparisons with SpCas9 in human cells revealed lower off-target mutagenesis levels, but also levels of on-target activity were reduced (Lee et al. 2016). Recently an ‘‘off-switch’’ system was found for NmCas9, which provides a means for invading bacteriophages to escape their bacterial host’s defence machinery (Pawluk et al. 2016). Three different proteins bind directly to the nuclease, preventing recognition of its target site and thus inhibiting DNA cleavage. These proteins were found to be active against NmCas9 in human cells, thus providing a method of tempering the activity of the enzyme.

### Francisella novicida Cas9 (FnCas9)

The Cas9 nuclease from *Francisella novicida* (FnCas9) is one of the largest Cas9 orthologues (1629 amino acids) and demonstrates an interesting feature; In addition to its conventional function as a nuclease in combination with crRNA:tracrRNA, FnCas9 can regulate target mRNA by association with a small CRISPR/Cas-associated RNA (scaRNA):tracrRNA complex (Sampson et al. 2013). This scaRNA, encoded within the Cas cluster, mediates its abrogation of target transcript by a mechanism which is independent of the RuvC and NHN endonuclease domains. The presence of scaRNA-like sequences in Cas gene clusters of several species suggests this non-canonical pathway for Cas9 could be more widespread. Investigations of the FnCas9’s nuclease function with substrates in vitro revealed specificity for an NGG PAM sequence (Hirano et al. 2016a). Interestingly, when applied in mammalian cells, no evidence of indel formation could be found. However, microinjection of mouse zygotes with FnCas9 as a ribonucleoprotein complex, led to highly efficient site-specific indel formation (Hirano et al. 2016a). Similar to previous work with SpCas9, structural information has facilitated its mutagenesis to generate a functional variant FnCas9 that recognizes an alternative 5′-YG-3′ PAM (E1369R/E1449H/R1556A substitutions, RHA FnCas9 variant), thus expanding the target space for this nuclease (Hirano et al. 2016a).

### Campylobacter jejuni Cas9 (CjCas9)

A new Cas9 orthologue derived from *Campylobacter jejuni* (CjCas9) was used successfully for genome editing in mammalian cells in vitro and in vivo (Kim et al. 2017a). Being one of the smallest Cas9 orthologues (984 amino acids, Fig. 2), a GFP-tagged CjCas9 was able to be packaged together with a expression cassette for its sgRNA in a single AAV vector and administrated to mice. Efficient indel mutagenesis was observed at the target loci in both muscle and the retina, suggesting potential therapeutic application of this Cas9 orthologue could be feasible. Kim et al. (2017a) also determined that CjCas9 recognizes a 22-nucleotide target sequence upstream of the PAM motifs 5′-NNNNACAC-3′ or 5′-NNNNRYAC-3′. A direct comparison of cutting efficiencies and off-target mutagenesis revealed that CjCas9 showed similar cutting efficiencies to SpCas9 and SaCas9, but CjCas9 was found to be more specific than SpCas9 and SaCas9 at a number of different target sites.

The study of the CjCas9 crystal structure in complex with an sgRNA and its target DNA complex reveals new insights into the function of this Cas9 orthologue. CjCas9, unlike other cas9 orthologues, bound both the target and non-target DNA strand (Yamada et al. 2017). Furthermore, the structure of the crRNA:tracrRNA was found to be substantially different to other Cas9 orthologues with the tracrRNA containing a triple-helix structure. These observations reveal once again the mechanistic diversity of the CRISPR-Cas9 systems.

## Cas12a (Cpf1)

The discovery of an additional class 2 CRISPR effector, originally described as Cpf1 and now reclassified as Cas12a (Shmakov et al. 2017), was received with great interest, mainly because the protein is structurally distinct from Cas9 effectors and possesses new interesting traits. Firstly, Cas12a binds to its target sequence by its association with a single RNA species, without the requirement of an additional tracrRNA (Zetsche et al. 2015a). Secondly, the Cas12a-crRNA complex recognizes a T-rich PAM sequence, lying 5′ of its target sequence (Fonfara et al. 2016; Yamano et al. 2016; Zetsche et al. 2015a), in contrast to the G-rich PAM sequence of the Cas9 systems that lies 3′ of its target. The T-rich PAM is of note as it might facilitate genome engineering in organisms with particularly AT-rich genomes. The PAM motif was initially reported as being TTTN; however, recent more in depth investigations in mammalian cells have revealed that target sequences with a TTTT PAM motif are inefficiently cleaved and this study redefined the Cas12a PAM as being TTTV (Kim et al. 2017b). Thirdly, Cas12a cleaves DNA via a staggered DSB, leaving a 4 or 5-nt 5′ overhang (Fonfara et al. 2016; Zetsche et al. 2015a), an attribute which might facilitate the introduction of specific sequences into the genome. Moreover, the staggered cleavage site of Cas12a from *Francisella novicida* U112 occurs after the 18th base on the non-targeted (+) strand and after the 23rd base on the targeted (–) strand, which is quite distant from the PAM sequence (Zetsche et al. 2015a). This distal cleavage, far away from both the seed region and the PAM, might preserve the target sequence for subsequent rounds of cleavage. This might be useful for encouraging repair via HDR for the targeted integration of exogenous DNA, since indel mutations caused by the dominant NHEJ repair pathway would be less likely to destroy the target site. Cas12a, orthologues of which have been identified in several bacterial and archeal genomes (Makarova et al. 2015), contains only a RuvC-like endonuclease domain and lacks the HNH domain present in Cas9 proteins. A putative novel nuclease domain, Nuc domain, has been ascertained from the crystal structure, which provides the 2nd nuclease activity responsible for cleaving the target strand (Yamano et al. 2016). Furthermore, a ribonuclease activity has been ascribed to this enzyme and has a role in processing of the precursor CRISPR RNA (Fonfara et al. 2016).

Screening of Cas12a-family enzymes from diverse bacteria identified two candidates from *Acidaminococcus* sp. BV3L6 (AsCas12a) and *Lachnospiraceae* bacterium ND2006 (LbCas12a) that are capable of mediating genome editing in human cells (Kim et al. 2016a; Zetsche et al. 2015a). Side-by-side comparisons of activity by measuring indel formation (Kim et al. 2016a) or assessing rates of homology directed repair efficiencies (Toth et al. 2016) revealed, in general, a lower activity for Cas12a orthologues when compared to SpCas9. However, the genome-wide analysis of cleavage sites in vitro for LbCas12a and AsCas12a showed that fewer off-target events were reported in comparison with Cas9 nucleases. Indeed, crystal structures of Cas12a-crRNA complexed with its DNA target reveal a unique mechanism for target recognition that is quite distinct from that reported for Cas9, which perhaps contributes to the increased specificity (Gao et al. 2016). However, it should be determined whether this increased specificity simply results from the smaller number of TTTV sites, the preferred PAM motif, in the mammalian genome or from the Cas12a system per se.

The potential of Cas12a for targeted mutagenesis in whole organisms was demonstrated by successful production of mutant mice by delivering AsCas12a and LbCas12a to the zygote by electroporation (Hur et al. 2016) and microinjection (Kim et al. 2016b; Watkins-Chow et al. 2017). Investigations of off-target mutagenesis by deep sequencing in the resulting mutant mice confirmed the high fidelity of Cas12a, with no mutations found at homologous sites (Hur et al. 2016; Kim et al. 2016b). On the other hand, a high level of mosaicism was observed in live pups after AsCas12a microinjection (Watkins-Chow et al. 2017).

## Cas13a (C2C2)

Further class 2 type CRISPR effectors have been mined from bacterial sequence databases using sophisticated computational pipelines, using the Cas1 sequence as an anchor to identify candidate loci (Shmakov et al. 2015). Using this strategy, two RuvC containing proteins distantly related to Cas12a (Cpf1), C2c1, now known as Cas12b, and C2c3, now known as Cas12c, were identified and were found to be present in diverse bacterial genomes. An additional protein family, originally named C2c2 and now classified as Cas13a (Shmakov et al. 2017), was identified (Shmakov et al. 2015) which, as confirmed by recent structural studies (Liu et al. 2017), contains 2 HEPN RNase domains, suggesting its target molecule could be an RNA. Expression of both Cas13a from *Leptotrichia shahii* (LsCas13a) and a crRNA against a target within the MS2 ssRNA bacteriophage in *E. coli* protected the bacteria from infection (Abudayyeh et al. 2016), confirming the minimal requirements for an operational RNA-guided site-specific RNAse.

Cas13a is directed to its target RNA by a single crRNA, similar to Cas12a and has preference for sequence with C, A and U immediately flanking the 3′ end of the protospacer. For activity in vitro, the crRNA requires target homology and is also dependent upon the secondary structure of the crRNA direct repeat stem. Surprisingly, in addition to the cleavage of the target RNA, it was found that LsCas13a, once activated, gains a non-specific RNAse activity and thus cleaves collateral RNA, ultimately leading to programmed cell death or senescence.

Although not yet applied in mammalian cells, functionality of the system was demonstrated for *Leptotrichia buccalis* Cas13a (LbCas13a) in Hela cell extracts (East-Seletsky et al. 2016). In this study, the non-specific RNAse activity of the activated enzyme was used to signal the presence of a specific target RNA in a population that activated the enzyme. Thus, although the promiscuous cleavage of RNAs might limit its use as a specific tool for ablating specific RNAs, this effector looks set to become a useful biotechnological tool for detecting specific RNAs, as has recently been demonstrated (Gootenberg et al. 2017). A greater understanding of how these RNAse activities work in consort to achieve site-specific cleavage of target RNAs may facilitate the application of this enzyme class as an experimental tool to ablate specific RNA transcripts in the absence of genomic cleavage. In addition, a catalytically inert Cas13a could be a useful device for the localization and imaging of RNA populations within the cell.

## Summary

In the few years since the discovery of SpCas9 and its first application in mammalian cells, we now have a wealth of enzymes with a range of activities and specificities. Mining the bacterial sequence databases has been lucrative and has provided a variety of novel enzymes. Where nature cannot provide the diversity of function needed, structural studies driving mutagenesis of the enzymes provides a means of tailoring specific characteristics to increase fidelity or broaden target range. It is hoped that the diversity of functions, which all these orthologues and variants provide, will facilitate therapeutic applications of these site-specific nucleases. As well as nucleases, these molecules can serve as effective DNA-binding domains, tethering machinery for visualizing specific loci or modulating gene expression (Dominguez et al. 2016). Indeed, engineered orthologues have already been used for both gene activation (Nishimasu et al. 2015) and for fluorescent labelling of genomic loci (Chen et al. 2016; Ma et al. 2015). The use of different deactivated CRISPR effectors recognizing varied sequences on DNA and even RNA will open up new possibilities for interrogating the genome and for future therapeutics.
